# The Influence of Childhood Adversity on Academic Performance, Cognitive Function, and Life Satisfaction in University Students

**DOI:** 10.1192/j.eurpsy.2025.1096

**Published:** 2025-08-26

**Authors:** K. K. Al Qassabi

**Affiliations:** Psychiatry, Oman Medical Specialty Board, Muscat, Oman

## Abstract

**Introduction:**

Childhood is a pivotal developmental phase, with experiences during this period significantly shaping future outcomes. Adverse Childhood Experiences (ACE), such as abuse, neglect, and household dysfunction, are linked to long-term adverse health outcomes, risky behaviors, and impaired cognitive function. These experiences can also negatively impact academic performance and life satisfaction. Despite the prevalence of (ACE), there is limited research in the Middle East.

**Objectives:**

The study aim to examines the relationship between Adverse Childhood Experiences (ACE), academic performance, cognition, and life satisfaction among Sultan Qaboos University students.

**Methods:**

This cross-sectional study recruited undergraduate students from Sultan Qaboos University using self-administered surveys distributed via social media and college administrations. It assessed ACE scores, academic performance (GPA), cognitive function, and life satisfaction through validated instruments.

**Results:**

The study found that 26.5% of students had high ACE scores, with emotional abuse being the most common. Higher ACE scores were associated with a history of mental illness, urban residence, and lower family financial status. Significant negative correlations were found between ACE scores and life satisfaction, GPA, and cognitive function. Regression analysis revealed that higher ACE scores increased the likelihood of lower GPA and decreased life satisfaction, emphasizing the substantial impact of ACEs on university students’ well-being and academic performance.

Table 1: Multinomial Logistic Regression Results of GPA as Dependent Factor.

**Table:** 

GPA Category	Predictor	B	Sig. (p-value)	Exp(B)	95% CI for Exp(B)
<2.00	ACE Score	0.317	0.025	1.373	1.041 - 1.811
	Gender	-1.639	0.001	0.194	0.073 - 0.517
	College	-0.229	0.02	0.795	0.656 - 0.964
2.00-2.49	Gender	-1.889	<0.001	0.151	0.066 - 0.345
	Academic Year	0.338	0.012	1.403	1.079 - 1.824
	College	-0.181	0.021	0.835	0.716 - 0.973
2.50-2.99	Academic Year	0.343	0.007	1.409	1.100 - 1.805

Table 2: multiple linear regression analysis SWLS as dependent variable.

**Table:** 

Predictor	B	Std. Error	Beta	t	Sig.	95% CI B
Gender	-0.932	0.577	-0.069	-1.614	0.107	-2.066 to 0.203
College	0.358	0.117	0.131	3.066	0.002	0.129 to 0.588
Financial Status	1.14	0.566	0.094	2.015	0.044	0.028 to 2.251
Diagnosed with Mental Illness	-3.355	1.015	-0.144	-3.305	0.001	-5.350 to -1.360
ACE Score	-1.112	0.174	-0.298	-6.41	0.00	-1.453 to -0.771

**Conclusions:**

The Study highlights the need for targeted interventions and support systems to address the diverse needs of students affected by (ACE). By understanding the long-term consequences of (ACE) and the mediating role of sociodemographic factors, educators, policymakers, and mental health professionals can develop strategies to promote resilience and well-being among young adults.

**Disclosure of Interest:**

None Declared

